# The cGMP-Dependent Protein Kinase 2 Contributes to Cone Photoreceptor Degeneration in the *Cnga3*-Deficient Mouse Model of Achromatopsia

**DOI:** 10.3390/ijms22010052

**Published:** 2020-12-23

**Authors:** Mirja Koch, Constanze Scheel, Hongwei Ma, Fan Yang, Michael Stadlmeier, Andrea F. Glück, Elisa Murenu, Franziska R. Traube, Thomas Carell, Martin Biel, Xi-Qin Ding, Stylianos Michalakis

**Affiliations:** 1 Department of Pharmacy—Center for Drug Research, Ludwig-Maximilians-University, 81377 Munich, Germany; MirjaKoch@gmx.de (M.K.); constanze.scheel@cup.uni-muenchen.de (C.S.); elisa.murenu@cup.lmu.de (E.M.); mbiel@cup.uni-muenchen.de (M.B.); 2 Department of Cell Biology, University of Oklahoma Health Sciences Center, Oklahoma City, OK 73104, USA; Hongwei-Ma@ouhsc.edu (H.M.); Fan-Yang@ouhsc.edu (F.Y.); xi-qin-ding@ouhsc.edu (X.-Q.D.); 3 Department of Chemistry, Ludwig-Maximilians-University, 81377 Munich, Germany; ms93@princeton.edu (M.S.); andrea.f.glueck@gmail.com (A.F.G.); franziska.traube@cup.lmu.de (F.R.T.); thomas.carell@cup.uni-muenchen.de (T.C.); 4 Department of Ophthalmology, Ludwig-Maximilians-University, 80336 Munich, Germany

**Keywords:** achromatopsia, cone photoreceptor, cGMP cytotoxicity, photoreceptor degeneration, neuroprotection

## Abstract

Mutations in the *CNGA3* gene, which encodes the A subunit of the cyclic guanosine monophosphate (cGMP)-gated cation channel in cone photoreceptor outer segments, cause total colour blindness, also referred to as achromatopsia. Cones lacking this channel protein are non-functional, accumulate high levels of the second messenger cGMP and degenerate over time after induction of ER stress. The cell death mechanisms that lead to loss of affected cones are only partially understood. Here, we explored the disease mechanisms in the *Cnga3* knockout (KO) mouse model of achromatopsia. We found that another important effector of cGMP, the cGMP-dependent protein kinase 2 (Prkg2) is crucially involved in cGMP cytotoxicity of cones in *Cnga3* KO mice. Virus-mediated knockdown or genetic ablation of *Prkg2* in *Cnga3* KO mice counteracted degeneration and preserved the number of cones. Analysis of markers of endoplasmic reticulum stress and unfolded protein response confirmed that induction of these processes in *Cnga3* KO cones also depends on Prkg2. In conclusion, we identified Prkg2 as a novel key mediator of cone photoreceptor degeneration in achromatopsia. Our data suggest that this cGMP mediator could be a novel pharmacological target for future neuroprotective therapies.

## 1. Introduction

Achromatopsia (ACHM) is an inherited retinal disorder affecting retinal cones, the type of photoreceptors that mediate high acuity daylight vision. Cone outer segments, the specialized compartments of these photoreceptors, contain all proteins needed for light detection and conversion into chemical and electrical signals. Mutations in genes encoding key proteins of this cascade result in total colour blindness, also referred to as achromatopsia. Approximately 80 percent of ACHM patients carry mutations in one of the genes *CNGA3* or *CNGB3* [1], which encode the two subunits of the cyclic nucleotide-gated (CNG) channel in cone photoreceptors [2]. The cone CNG channel is part of the visual transduction cascade located in the cone outer segment and is the effector of cyclic guanosine monophosphate (cGMP), the key second messenger of this signaling cascade, which translates light signals into electrical and Ca^2+^ signals [2]. Four additional disease genes exist, among which *GNAT2*, *PDE6C*, and *PDE6H* also encode proteins involved in the cone visual transduction cascade [1]. The sixth known disease gene is *ATF6*, encoding an endoplasmic reticulum (ER)-localized transmembrane transcription factor that can activate the unfolded protein response (UPR) and plays a role in ER homeostasis [3,4].

Like many other inherited disorders, ACHM manifests already in childhood with clinical symptoms that include lack of colour discrimination, poor visual acuity, extreme light sensitivity (photophobia), and involuntary eye movements (nystagmus) [5]. Given the lack of cone photoreceptor function from beginning, there is no real progression of the clinical symptoms over time. However, animal experiments and morphological data from affected patients suggested a progressive degeneration and loss of cones over time [6,7]. While the principal development and morphology of affected cone photoreceptors is initially similar to non-affected cones, the diseased cones start degenerating during young adulthood and are eventually lost by induction of various cell death mechanisms [8].

Currently, there is no treatment available for ACHM, but several groups are working on the development of gene supplementation therapies for both *CNGA3*- and *CNGB3*-linked ACHM [9]. One phase I/II clinical trial testing the effect of an adeno-associated virus (AAV)-based gene therapy vector in patients with confirmed *CNGA3*-linked ACHM was already completed and recently reported promising safety and efficacy data [10]. Three additional ACHM gene therapy clinical trials are ongoing and expected to report first data soon.

The concept of gene supplementation with AAV vectors is only applicable at early stages of the disease and providing a sufficient number of morphologically intact and, thus, rescuable cone photoreceptors is still present [9]. Given that affected ACHM cones degenerate over time, there is only a certain time window of opportunity for gene supplementation therapies. Unfortunately, the disease mechanisms involved in cone degeneration are only partially understood and there is a high need to better characterise the pathobiology in affected cone photoreceptors.

Here, we investigated the role of cGMP signalling on viability of cone photoreceptors in the *Cnga3* knockout model of ACHM. We found that the membrane bound cGMP-dependent protein kinase 2 (Prkg2) is crucially involved in cGMP-mediated cytotoxicity in cones. Genetic depletion of Prkg2 resulted in a long-lasting preservation of cone photoreceptors. Mechanistically this neuroprotective effect of Prkg2 depletion on cones seems to involve inhibition of DNA damage, unfolded protein and ER-stress mechanisms. Our work highlights the cGMP kinase Prkg2 as a novel target for neuroprotective treatments aiming to preserve the morphology and structure of affected ACHM cone photoreceptors.

## 2. Results

**Figure 1:**
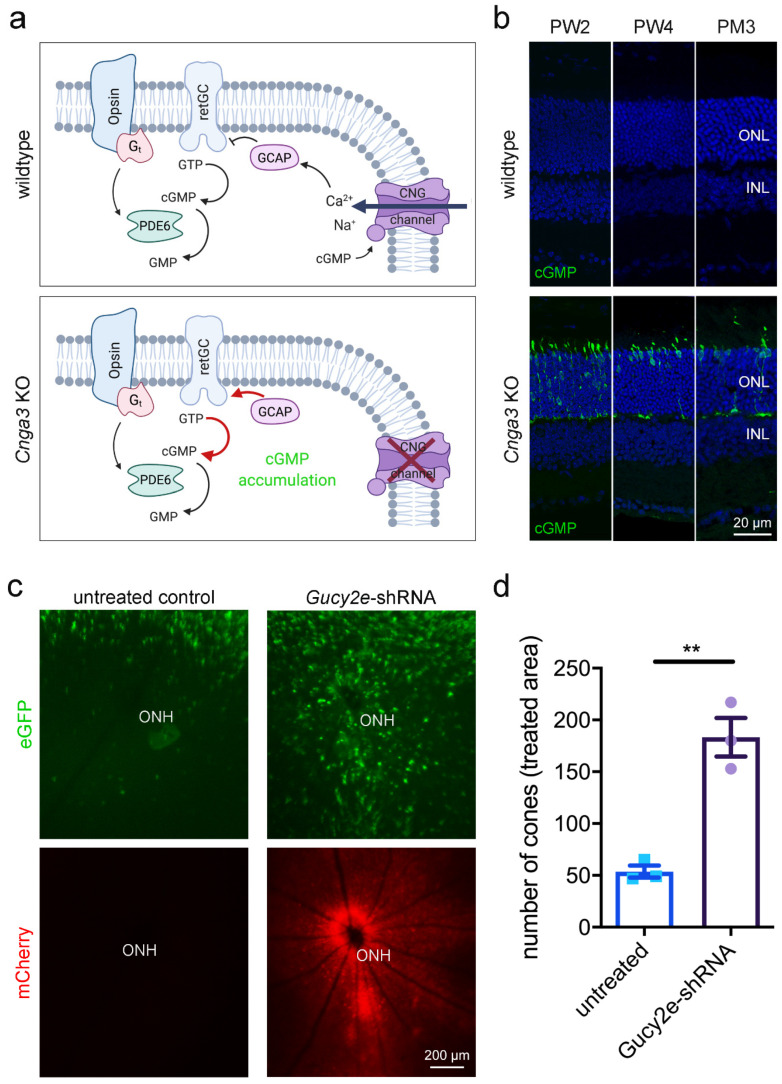
Accumulation of cyclic guanosine monophosphate (cGMP) in cone photoreceptors of *Cnga3* KO mice. (**a**) Cartoons illustrating key components of the visual signalling cascade in wildtype (upper part) and *Cnga3* KO (lower part) cone outer segments (see text for details). The cartoon was created with BioRender.com. (**b**) Confocal scans from wildtype (upper panels) and *Cnga3* KO (lower panels) retinal cross-sections immunolabelled for cGMP (green) to illustrate the exuberant levels of cGMP in affected cones. Due to the relatively low affinity, the anti-cGMP antibody fails to detect any signal in the wildtype retina. The scale bar applies to all images. (**c**,**d**) Subretinal administration of adeno-associated virus (AAV) vectors encoding a shRNA directed against *Gucy2e* preserves the number of cones in RG-eGFP/*Cnga3* KO mice. In addition to the *Cnga3* deletion, the mouse line carries a transgene for cone-specific expression of eGFP. The delivered AAV vector also mediates mCherry expression, which allows for visualisation of the virally transduced area in the fundus imaging (**c**). The contralateral non-injected eye served as control. The scale bar applies to all images. (**d**) Quantification of the number of eGFP-positive cones from fundus fluorescence images revealed a significant preservation upon AAV-*Gucy2e*-shRNA treatment at 3 months after injection (*n* = 3, ** *p* < 0.01, Student’s *t*-test). Error bars shown are SEM. INL, inner nuclear layer; ONH, optic nerve head; ONL, outer nuclear layer; PW, postnatal week.

In cone outer segments, the second messenger cGMP is produced by the receptor guanylyl cyclase (retGC encoded by the *GUCY2E* gene) and its levels are balanced by the cone phosphodiesterase (PDE6C; Figure 1a). High levels of cGMP in the dark activate the cyclic nucleotide-gated (CNG) channel, which carries a mixed Na^+^ and Ca^2+^ inward current. Ca^2+^-bound guanylyl cyclase activating protein (GCAP1) inhibits the retGC activity in a negative feedback mechanism. In cones missing the CNG channel, the lack in Ca^2+^ influx would likely determine the constant activation of retGC by Gcap1 and the production of excessive amounts of cGMP (Figure 1a). Indeed, immunolabeling of retinal cross-sections from *Cnga3* KO mice, which lack the cone CNG channel [6], with a cGMP-specific antibody [11] confirmed the accumulation of cGMP in cone photoreceptors (Figure 1b) at eye opening and persisting at later timepoints. In parallel, the number of cGMP-positive cones decreased over time, reflecting the already described progressive cone degeneration in *Cnga3* KO mice [6,8]. Double labelling for cGMP and the cone marker glycogen phosphorylase confirmed that the cells showing high levels of cGMP are cone photoreceptors (Supplementary Figure S1). This is also the case for those cGMP-positive cells localising in the lower part of the outer nuclear layer.

To investigate the effect of acute downregulation of retGC, we generated an adeno-associated virus (AAV) vector encoding a shRNA that targets the endogenous *Gucy2e* mRNA and we delivered it via subretinal injection into the eyes of 2-week-old *Cnga3* KO mice expressing an eGFP reporter under the control of the cone-specific human red/green opsin (RG) promoter [12]. This treatment resulted in preservation of a higher number of cones in areas of *Gucy2e* downregulation (Figure 1c,d). This is in line with a previous report showing that constitutive genetic inactivation of *Gucy2e* in *Cnga3* KO mice delayed degeneration and preserved biochemical markers of cone photoreceptors [13].

**Figure 2:**
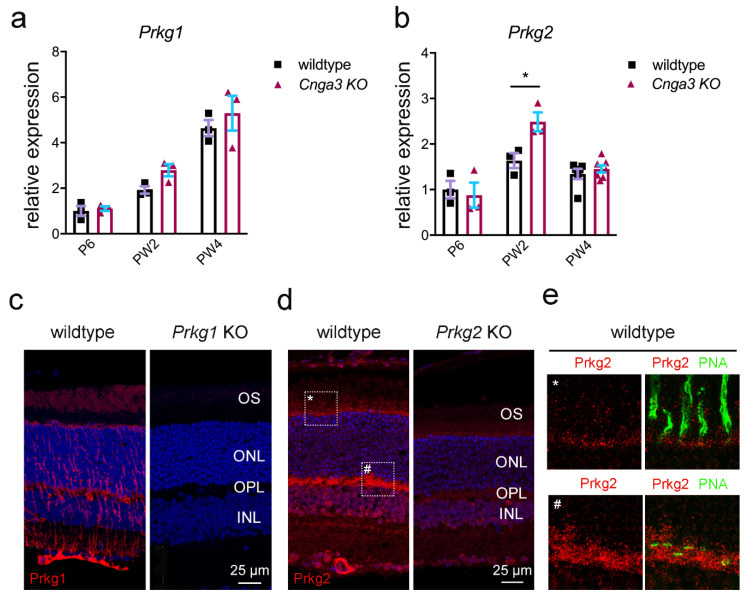
Expression of the cGMP dependent kinases Prkg1 and Prkg2 in wildtype and *Cnga3* KO retina. (**a**,**b**) Relative *Prkg1* (**a**) and *Prkg2* (**b**) transcript levels at indicated time points. While *Prkg1* is expressed at similar levels in wildtype and *Cnga3* KO retina (**a**), there is a transient significant upregulation of *Prkg2* transcript in the *Cnga3* KO retina around eye opening (PW2) (**b**, *n* = 3–6, * *p* < 0.01, 1-way-ANOVA). (**c**–**e**) Immunolocalization signal for Prkg1 (**c**) and Prkg2 protein (**d**,**e**) in the mouse retina. (**c**) The intense Prkg1 signal (red) is mostly confined to Müller glia cells and blood vessels with additional faint signal in photoreceptor outer segments (OS). (**d**) Intense Prkg2 signal is found in photoreceptor inner segments (see higher magnification view on marked regions in panel (**e**)), synapses in the outer plexiform layer (OPL), some inner nuclear layer (INL) cells and in blood vessels. Panels **c** and **d** show Hoechst nuclear dye signal in blue and panel E shows the cone marker peanut agglutinin (PNA) signal in green. Tissue from the corresponding knockout mouse lines (*Prkg1* KO in C and *Prkg2* KO in (**d**)) did not produce a signal similar to the wildtype. The scale bar in **c** and **d** applies to both images, respectively. (**e**) Magnification view of the corresponding regions (*,#) marked with dotted rectangles in the left (wildtype) image of (**d**).Error bars shown are SEM. ONL, outer nuclear layer; P, postnatal day; PW, postnatal week.

The key effector of cGMP in cone photoreceptors is the CNG channel [2]. Since the CNG channel is absent from *Cnga3* KO cones, we explored the expression of the cGMP-dependent kinases, namely Prkg1 and Prkg2. As shown in Figure 2, both kinases are expressed in the mouse retina. When analysing the transcript levels in *Cnga3* KO retina at different postnatal time points, we observed a transient upregulation of *Prkg2* transcript on week 2 (Figure 2b), whereas *Prkg1* did not reveal any genotype-specific changes (Figure 2a).

We then analysed the immunolocalization of the two kinases on retinal cross-sections. The Prkg1 signal was primarily found in Müller glia cells and retinal blood vessels and only a faint signal could be detected in the outer segment layer of photoreceptors (Figure 2c). Importantly, this signal was absent in cross-sections from *Prkg1* knockout animals (Figure 2c). Prkg2 showed a distinct expression pattern with strong labelling of retinal blood vessels, but also of the photoreceptor synaptic layer (outer plexiform layer, OPL) and weaker labelling of the photoreceptor inner segments (Figure 2d,e for high magnification view). While the Prkg2 antibody signal was not completely absent in tissue from *Prkg2* KO mice, it was substantially reduced compared to the wildtype condition (Figure 2d).

Given the exuberant accumulation of cGMP in *Cnga3* KO cones and the transient upregulation of the potential effector Prkg2, we wondered whether this kinase plays any role for the survival of affected cones. We, therefore, generated an AAV vector encoding a shRNA that targets the *Prkg2* mRNA and delivered it via subretinal injection into the eyes of 2-week-old RG-eGFP/*Cnga3* KO mice.

**Figure 3:**
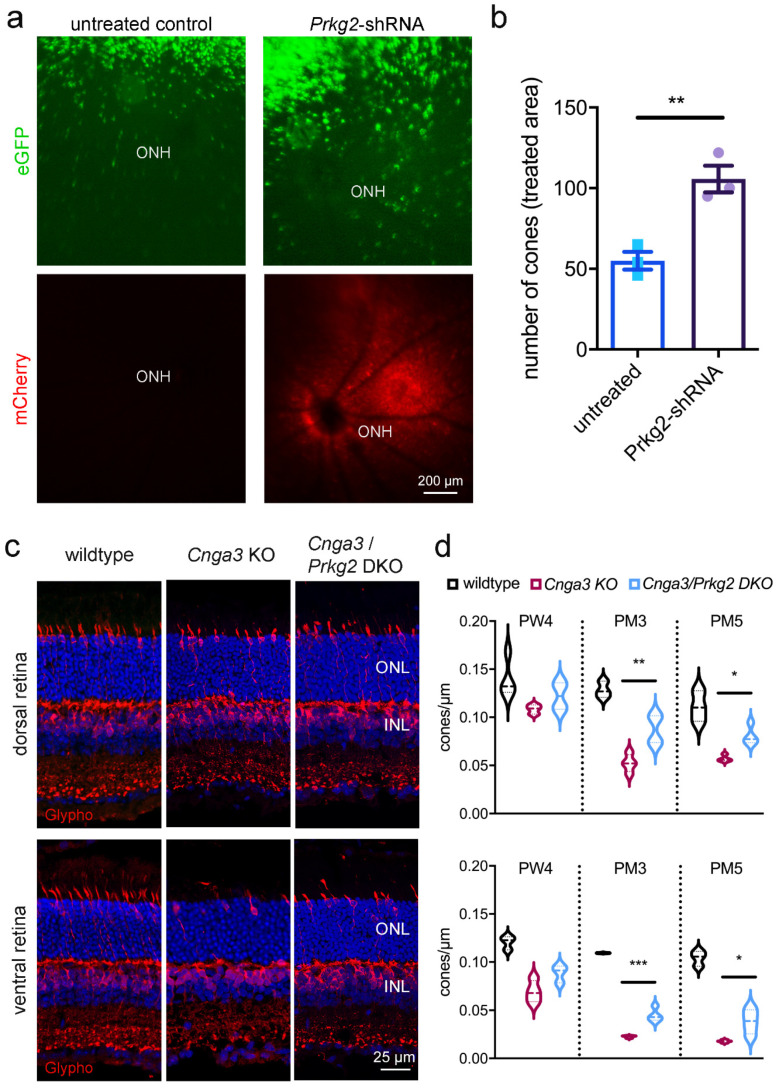
Downregulation or knockout of Prkg2 delays cone degeneration in *Cnga3* KO mice. (**a**,**b**) Subretinal administration of AAV vectors encoding a shRNA directed against *Prkg2* preserves the number of cones (green) in RG-eGFP/*Cnga3* KO mice. The retinal area targeted by the subretinal injection of the AAV vector is indicated by the fundus fluorescence signal of the vector-encoded mCherry (red) (lower panel in **a**). The scale bar applies to all images. (**b**) Quantification of the number of eGFP-positive cones from fundus fluorescence images revealed a significant preservation upon AAV-*Prkg2*-shRNA treatment at 3 months after injection (*n* = 3, ** *p* < 0.01 Student’s *t*-test). Error bars shown are SEM. ONH, optic nerve head. (**c**,**d**) Genetic inactivation of Prkg2 preserves *Cnga3*-deficient cone photoreceptors. (**c**) Cone morphology in wildtype, *Cnga3* KO and *Cnga3/Prkg2* double knockout (DKO) retina visualized by immunolabeling of glycogen phosphorylase (glypho). The anti-glypho signal is found in cone inner segment, cell body and synapse. In addition, anti-glypho labels bipolar cell bodies and synapses in the outer nuclear layer (ONL) and inner plexiform layer. Panels in C show Hoechst nuclear dye signal in blue. The scale bar applies to all images. (**d**) Violin plot showing quantification of cone numbers from glypho labelled retinal cross-sections revealing a significantly higher density of cones in *Cnga3/Prkg2* DKO compared to *Cnga3* KO (*n* = 3–4, * *p* < 0.05, ** *p* < 0.01, *** *p* < 0.005, 1-way-ANOVA). INL, inner nuclear layer; PM, postnatal month; PW, postnatal week.

This treatment led to significant preservation of the number of cones at 3 months of age (Figure 3a,b), suggesting that the *Prkg2* knockdown has a neuroprotective effect on *Cnga3* KO cones. To corroborate this finding, we cross-bred *Cnga3* KO mice with mice lacking Prkg2 [14] and analysed the survival of cone photoreceptors in the resulting *Cnga3*/*Prkg2* double knockout (DKO) as well as in *Cnga3* KO and wildtype mice (Figure 3c,d). To determine the cell density of cones, we immunolabeled retinal cross-sections from these mice for glycogen phosphorylase, which, in addition to bipolar cells, labels the entire cytoplasm of cones from the inner segment throughout the synapse and thus facilitated quantification [15]. While at 4 weeks of age the cone density did not substantially differ among genotypes, the number of cones was significantly higher in *Cnga3*/*Prkg2* DKO than in *Cnga3* KO mice at 3 and 5 months of age (Figure 3d). This was evident in the dorsal retina, but also in the faster degenerating ventral part (Figure 3d). Thus, constitutive knockout or acute knockdown of *Prkg2* protected Cnga3 KO cone photoreceptors from degeneration.

**Figure 4:**
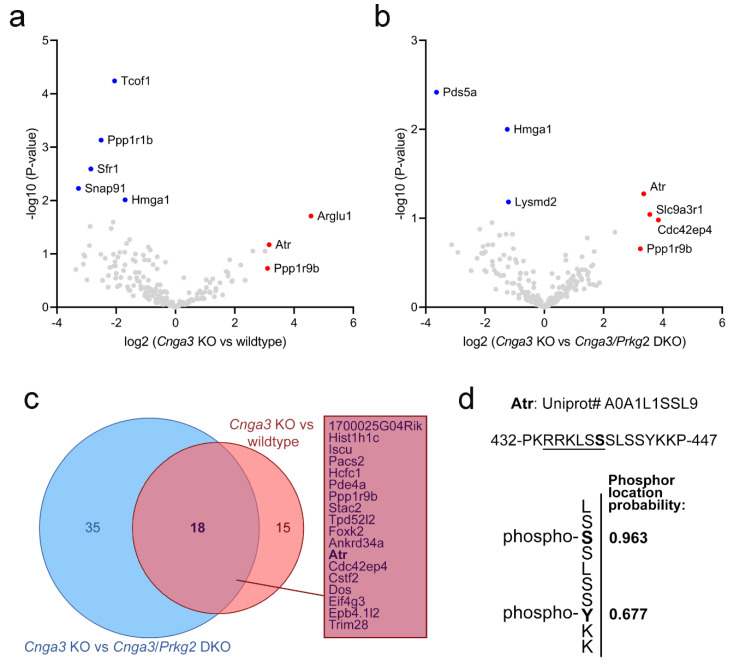
Mass spectrometry-based analysis of Ti-IMAC-enriched phosphoproteins in mouse retinal lysates from *Cnga3* KO mice. (**a**,**b**) Volcano plots showing the enrichment/depletion of identified phosphoproteins in *Cnga3* KO vs. wildtype (**a**) or versus *Cnga3*/*Prkg2* (**b**) DKO retinal lysates at postnatal week 4 (each dot represents a specific identified phosphorylated peptide). (**c**) Venn diagram showing the number of hits that were enriched in *Cnga3* KO over wildtype (blue) or *Cnga3*/*Prkg2* DKO (red). The 18 hits enriched in both comparisons are listed in the rectangle. (**d**) Sequence of the identified Atr-derived peptide with information on the identified phosphorylation sites (see Supplementary Figure S2 for a representative mass spectrometry (MS) spectrum).

Since Prkg2 is a cGMP-dependent serine/threonine-specific kinase, it is tempting to speculate that this neuroprotective effect is mediated by the lack of its kinase activity. We, therefore, designed an experiment to identify potential changes in the pattern of phosphoproteins in *Cnga3* KO mice. In particular, we used titanium ion (Ti^4+^) functional magnetic microparticles (Ti-IMAC) to enrich phosphorylated peptides [16] from wildtype, *Cnga3* KO, and *Cnga3*/*Prkg2* DKO retinal protein lysates for subsequent label free quantification (LFQ) using mass spectrometry (Figure 4). We reasoned that relevant proteins whose phosphorylation pattern depends on elevated activity of Prkg2 would show an enrichment in *Cnga3* KO over wildtype retina and a depletion from *Cnga3*/*Prkg2* DKO retinal phospho-lysates. As visualized by the volcano plots in Figure 4a,b, several proteins were found to be enriched in phospho-lysates of *Cnga3* KO retina compared to wildtype or *Cnga3*/*Prkg2* DKO. Among a total of 18 proteins showing the aforementioned enrichment/depletion pattern, we found the serine/threonine-specific protein kinase Atr (Atm-Rad3-related protein). Phosphorylated Atr peptides were found in *Cnga3* KO retinal lysates, but were not detected in lysates from *Cnga3*/*Prkg2* DKO or wildtype retina.

The MaxQuant analysis software [17] allows for determination of the most likely phosphorylation locations in the identified phosho-peptides. For the Atr peptides, a high phospho location probability of 0.963 was determined for serine 439 (Ser439) and a localisation probability of 0.677 for tyrosine 444 (Y444). An example mass spectrometry (MS) spectrum showing fragments used for identification and phosphor-localization is depicted in Supplementary Figure S2.

**Figure 5:**
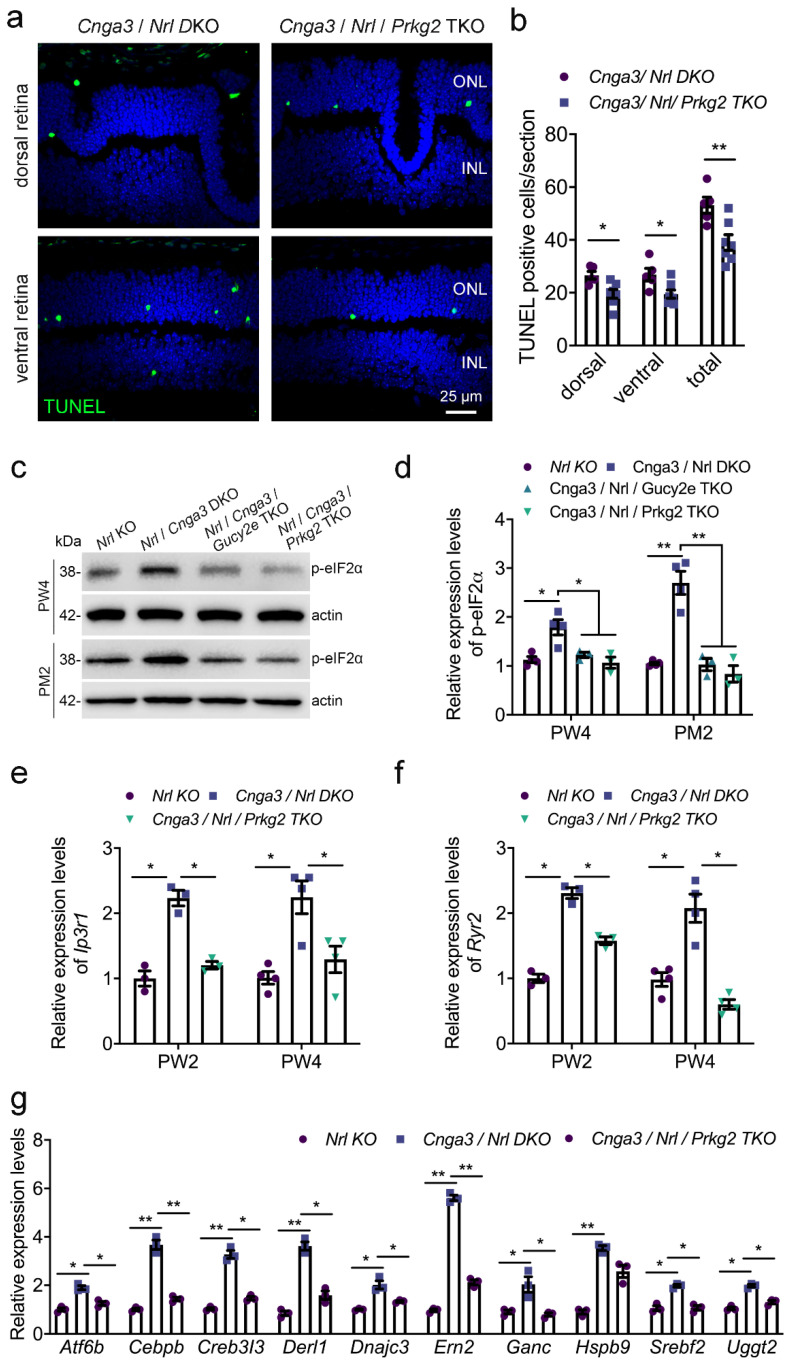
Dependence of Cnga3-linked ER stress and unfolded protein response (UPR) on Prkg2. (**a**,**b**) TUNEL-based evaluation of cell death on retinal cross sections of *Nrl* KO, *Cnga3*/*Nrl* DKO, and *Cnga3*/*Nrl*/*Prkg2* TKO mice at week 2. Shown are representative confocal images of TUNEL labeling (**a**) and the corresponding quantifications (**b**, *n* = 6–8). The scale bar in (**a**) applies to all shown images. (**c**,**d**) Western blot analysis of the ER stress marker p-eIF2a in retinal lysates from *Nrl* KO, *Cnga3*/*Nrl* DKO, *Cnga3*/*Nrl*/*Gucy2e* TKO, and *Cnga3*/*Nrl*/*Prkg2* TKO mice at postnatal month (PM) 1 and 2. Shown are representative immunoblotting images (**c**) and the corresponding quantifications (**d**, *n* = 8−10). (**e**–**g**) mRNA expression levels of *Itpr1* (**e**), *Ryr2* (**f**) and indicated UPR-related genes in retinas of *Nrl* KO, *Cnga3*/*Nrl* DKO, and *Cnga3*/*Nrl*/*Prkg2* TKO mice at week 2 and 4 (**e**,**f**, *n* = 8–10) or at week 2 (**g**, *n* = 6–8). * *p* < 0.05, ** *p* < 0.01, 1-way-ANOVA. Error bars shown are SEM. INL, inner nuclear layer; ONL, outer nuclear layer; PM, postnatal month; PW, postnatal week.

It is well established that increased ER stress contributes to degeneration and cell death of *Cnga3* KO cone photoreceptors [18,19,20]. Thus, we next evaluated how *Prkg2*-deletion influences ER stress markers in *Cnga3* KO cones. To facilitate the bulk analysis of cone photoreceptor markers, we generated a *Cnga3*/*Prkg2* double-deficient mouse line on the cone-dominant *Nrl* KO background [20]. *Cnga3*/*Nrl* DKO mice display a cone degeneration similar to the *Cnga3* single KO [13,20]. We first compared the level of photoreceptor cell death in *Cnga3*/*Nrl* DKO and *Cnga3*/*Nrl*/*Prkg2* triple knockout (TKO) mice. As shown in Figure 5a,b, deletion of *Prkg2* lead to a significant reduction of the Terminal deoxynucleotidyl transferase dUTP nick end labelling (TUNEL) signal, indicative of a reduced cell death rate. We next analyzed the effect of Prkg2 knockout on the levels of phosphorylation of eukaryotic initiation factor 2 (p-eIF2a) which is an established ER stress marker already shown to be upregulated in *Cnga3*-deficient cone photoreceptors [20]. In line with a neuroprotective effect of *Prkg2*-deletion, the levels of p-eIF2a were reduced to a level similar to *Cnga3*/*Nrl*/*Gucy2e* TKO mice (Figure 5c,d), which are protected from cGMP-mediated cytotoxicity due to the lack of the cGMP-synthesizing retGC enzyme [13]. Previous studies have demonstrated that CNG channel-deficient mice show increased expression/activity of ER Ca^2+^-releasing channels, responsible for Ca^2+^ efflux from the ER into the cytosol [20,21]. We, therefore, analyzed the gene expression of *Itpr1*, which encodes the inositol 1,4,5-trisphosphate receptor type 1 (IP3R1), and *Ryr2*, the gene encoding the type 2 ryanodine receptor (Ryr2). As depicted in Figure 5e,f, both genes showed enhanced gene expression levels in the *Cnga3* KO context, which were normalized after additional knockout of *Prkg2*. A similar effect was observed for the unfolded protein response (UPR)-related genes *Atf6b* (activating transcription factor 6 beta), *Bax* (Bcl2-associated X protein), *Cebpb* (CCAAT/enhancer-binding protein beta), *Creb3l3* (cAMP-responsive element-binding protein 3-like 3), *Derl1* (degradation in ER protein 1), *Dnajc3* (DnaJ [Hsp40] homolog, subfamily C, member 3), *Ern2* (ER to nucleus signaling 2), *Ganc* (glucosidase, alpha; neutral C), *Srebf2* (sterol regulatory element-binding factor 2), and *Uggt2* (UDP-glucose glycoprotein glucosyltransferase 2; Figure 5e–g), which are upregulated in *Cnga3*-deficient mice [19,21]. This suggests that *Prkg2* knockout counteracts ER stress and UPR and normalizes ER homeostasis.

## 3. Discussion

Loss of vision is one of the most severe handicaps with high socioeconomic importance. Many blinding disorders are inherited and caused by mutation in one of more than 250 retinal disease genes (https://sph.uth.edu/retnet/), which affect rod and cone photoreceptors function. Until recently, there was no hope for patients suffering from blinding inherited retinal disorders. This has changed with the approval of a first gene therapy for a specific condition, *RPE65*-linked retinal dystrophy [22]. Many more of these gene-specific treatments or more broadly applicable therapies are needed in order to cover all conditions caused by the >250 disease genes.

In addition to a loss of function, a degeneration of affected photoreceptors is commonly observed. In order to provide a long-lasting treatment effect, any therapy needs to halt the degeneration processes. This is true for disorders like retinitis pigmentosa, which are a class of inherited retinal dystrophies characterized by a progressive degeneration of rod photoreceptors. It also applies to conditions like achromatospia affecting cone photoreceptors and, thus, high acuity daylight vision. However, the mechanisms involved in rod and cone degeneration are only partially understood and a more complete understanding is needed to develop improved treatments [8,23,24].

Here, we explored cone cell death mechanisms in the *Cnga3* KO mouse model of achromatopsia. *Cnga3* KO cone photoreceptors lack the ability to respond to light stimuli [25] and cannot generate or transfer corresponding signals to higher order neurons and central visual centres. In addition to the loss of function, cones are degenerating over time, inducing ER stress and eventually dying [6,8,19,20]. Affected cone photoreceptors also accumulate exuberant levels of cGMP due to lack of the cone CNG channel and, thus, absence of the Ca^2+^-mediated negative feedback inhibition of the cGMP-synthesizing retGC enzyme (see Figure 1A and corresponding text for more details). Deletion or knockdown of *Gucy2e* eliminates the cGMP accumulation and preserved the number of cones (Figure 1 and [13]), confirming that cGMP is crucially involved in the degeneration process.

The CNG channel itself is a major effector of cGMP and, if still present, was shown to be an important factor in mediating cGMP cytotoxicity [24,26]. However, *Cnga3* KO cones lack a cGMP-gated channel and, thus, cGMP cytotoxicity must be due to other cGMP effectors or an impairment of more generic cell homeostasis/metabolic processes. Other potential cGMP effectors are the cGMP-dependent serine/threonine kinases encoded by the two genes *Prkg1* and *Prkg2*, which are involved in a variety of physiological and pathophysiological processes, but have no established physiological function in retinal photoreceptors [27]. Indeed, the role of Prkg1 in survival of diseased rod photoreceptor has been already addressed [28,29]. Based on this, inhibition of Prkg1 is currently explored as a neuroprotective treatment to delay degeneration of photoreceptors in retinitis pigmentosa [28,30]. In contrast, the role of Prkg2 in photoreceptor degeneration has not been investigated so far.

Here, we discovered a crucial role of the Prkg2 in degenerating *Cnga3* KO cone photoreceptors. We found that AAV-mediated knockdown or genetic deletion of *Prkg2* rescues *Cnga3* KO cone photoreceptors from degeneration. While the effect on cone survival is clear, deletion of Prkg2 does not fully prevent all cone photoreceptors from dying. This suggests that, besides cGMP-Prkg2-mediated cytotoxicity, additional cell death mechanisms contribute to the degeneration of cones. Mechanistically, deletion of Prkg2 dampened the levels of ER stress and UPR markers, suggesting that cGMP-Prkg2 signalling contributes to both phenomena and crucially impacts viability of CNG channel-deficient cone photoreceptors [18,21,31].

Using an approach based on Ti-IMAC enrichment of phosphorylated proteins in combination with *Cnga3*/*Prkg2* DKO tissue as a reference, we were able to identify phosphoproteins that are upregulated in *Cnga3* KO retinal tissue and whose phosphorylation depends on Prkg2. In total we identified 18 phosphoproteins being enriched in *Cnga3* KO retina against both wildtype and *Cnga3*/*Prkg2* DKO. Among those we found Atr, which belongs to the phosphatidylinositol 3-kinase-related kinase family and is a known DNA damage sensor, activating the DNA damage response (DDR) by phosphorylating downstream effector proteins [32]. Of note, the MaxQuant analysis software identified Ser439 as the most likely phosphorylation site. Ser439 is part of a putative recognition motif (underlined sequence in Figure 4d) that is similar to the sequence of previously postulated Prkg2 substrates [33]. Given that the identified Ser439 phosphorylation has not been previously characterized, its relevance for the degeneration process in *Cnga3* KO cones remains unclear. At this stage we can only speculate that cGMP-Prkg2 signalling triggers cell death by activating DNA damage pathways. This intriguing hypothesis deserves further investigation in the future, in particular since recent evidence suggests a cross-talk between DDR and ER stress [34].

Further studies are also needed to get more insight into the timing of the pathophysiological events that eventually lead to cone cell death. From the upregulation of *Prkg2* transcript at PW2, we can speculate that a transient upregulation of the cGMP-Prkg2 signalling around eye opening could trigger subsequent cell death processes. Since the progression of cone degeneration in the *Cnga3* KO mouse is rather slow, we believe that multiple signaling processes running in parallel contribute to degeneration of cones; thus, Prkg2-dependent mechanisms are responsible only in part for the observed effects and do not dictate the kinetics of degeneration. Additionally, more focused experiments with *Cnga3* KO mice on a cone-rich *Nrl* KO background are needed to clarify this issue. 

Taken together, this study establishes Prkg2 as a novel therapeutic target for neuroprotection of degenerating cone photoreceptors in achromatopsia, although the therapeutic applicability of Prkg2 inhibition needs to be further validated. Another aspect that remains to be elucidated is whether this neuroprotective effect is specific for cones or if it is also relevant for rod photoreceptors. Moreover, Prkg2 inhibition could provide protection not only from cGMP-mediated cytotoxicity, but also from degeneration caused by other detrimental stimuli. Importantly, in the case of inherited retinal disorders like achromatopsia, a genetic mutation causes a functional defect. Thus, the underlying gene defect needs to be corrected or a healthy copy of the affected gene needs to be supplemented in order to achieve an improvement in cone function. However, such gene correction or supplementation therapies are only applicable if a sufficient number of morphologically intact and, thus, rescuable target cells remains. Therefore, advanced stage patients who lost the majority of their photoreceptors are excluded from gene-specific gene therapies. Based on the presented results it is tempting to speculate that a neuroprotective therapy by Prkg2 inhibition has the potential to delay degeneration of cones and to widen the window of opportunity for gene therapy in achromatopsia.

## 4. Materials and Methods

### 4.1. Animals

All animal experiments were performed according to the ARVO statement for the use of animals in ophthalmic and vision research and were approved by the local authorities (the Government of Upper Bavaria, Germany or the Institutional Animal Care and Use Committee of the University of Oklahoma Health Sciences Center, Oklahoma City, OK, USA). The project identification codes were ROB-55.2-2532.Vet_02-14-59 and 18-060-EAFI, respectively. All mice used were on a mixed C57-BL6/J-129/Sv genetic background. *Cnga3*/*Prkg2* double knockout mice were generated by cross-breeding *Cnga3* KO mice [25] with *Prkg2* KO mice [14]. *Cnga3*/*Nrl* DKO and *Cnga3*/*Nrl*/*Gucy2e* TKO mice were generated by cross-breeding as described previously [19,35]. *Cnga3*/*Nrl*/*Prkg2* TKO mice were generated by cross-breeding *Cnga3* KO mice with *Prkg2* KO mice [14] and/or *Nrl* KO [36] mice. For in vivo imaging of cones *Cnga3* KO mice were also crossbred with RG-eGFP mice [37] to generate RG-eGFP/*Cnga3* KO mice. Mice were maintained on a 12 h light/dark cycle. During the light cycle, cage illumination was approximately 7 foot-candles.

### 4.2. Eye Preparation, Immunofluorescence Labelling, and Confocal Microscopy

We prepared mouse eye cross-sections for immunohistochemical analysis as described previously [35,38]. Cell nuclei were stained with Hoechst 33,342 (Thermo Fisher Scientific, Langenselbold, Germany). The following primary antibodies were used: anti-cGMP (sheep, diluted 1:500, from Prof. Steinbusch, Maastricht University; anti-cGKI alpha (rabbit, 1:100, kindly rovided by F. Hofmann); anti-PRKG2 (rabbit, 1:50, Merck-Sigma Aldrich, Cat. Nr. HPA007386). In order to preabsorb against non-specific epitopes, the anti-PRKG2 antibody was preincubated for 3 h at 4 °C with brain and retina homogenate from a *Prkg2* KO mouse, then cleared by centrifugation and used at a final dilution of 1:50 for immunohistochemistry. Confocal images were collected at a Leica TCS SP8 spectral confocal laser scanning microscope (Leica Microsystems). The generated files were processed with LAS X software and the open-source software Fiji [39,40].

### 4.3. TUNEL Assay

The terminal deoxynucleotidyltransferase dUTP nick end-labelling (TUNEL) was performed and analysed as described previously [20,35]. Confocal images were collected at an Olympus FV1000 confocal laser scanning microscope (Olympus, Melville, NY, USA).

### 4.4. Cloning and Production of AAV Vectors

For shRNA experiments a modified pSub201 plasmid [41] was generated with a shRNA expression cassette and U6 promoter. Additionally, a phosphoglycerate kinase 1 (PKG) promoter-driven mCherry with SV 40 polyA and WPRE was inserted by standard restriction enzyme based cloning techniques. The resulting plasmid and shRNA hairpin sequences were pSub_U6_shRNAGucy2e_PGK_mcherry_SV40_WPRE, pSub_U6_shRNAPrkg2_PGK_mcherry_SV40_WPRE, and pSub_U6_shRNACtrl_PGK_mcherry_SV40_WPRE. Sequences are available upon request. AAV8-Y733F-pseudotyped AAV2 vectors carrying the corresponding shRNA/mCherry expression cassettes were produced, purified and characterized as previously described [38].

### 4.5. Subretinal Injection and In Vivo Fundus Fluorescence Imaging

1 µL of titer-matched AAV8-shRNA vector containing approx. 5E10 total vector genomes was delivered via subretinal injection in 2-week-old RG-eGFP/*Cnga3* KO mice. Fundus fluorescence examination was performed with an adapted Spectralis HRA + OCT system from Heidelberg Engineering (Dossenheim, Germany) in combination with optic lenses, as described previously [42].

### 4.6. Retinal Protein Preparation, SDS-PAGE, and Western Blot Analysis

Retinal protein preparation, SDS-PAGE, and western blot analysis were performed as described previously [21,35]. A Li-Cor Odyssey machine and Li-Cor software (Li-Cor Biosciences, Lincoln, NE, USA) were used for detection and densitometric analysis.

### 4.7. PCR and Quantitative RT-PCR

Total RNA preparation and reverse transcription were per- formed as described previously [19,21]. The quantitative reverse transcription-polymerase chain reaction (qRT-PCR) assays were performed with a real-time PCR detection system (iCycler; Bio-Rad, Hercules, CA, USA). The primers used and the ∆∆Ct method used are described in [19,21].

### 4.8. Ti-IMAC Phosphoenrichment and MS-Sample Preparation

After lysis, samples were incubated with 1 µL Benzonase for 1 h at 0 °C. Afterwards, 250 µL of MS-grade water was added and 2 retinas from the same date were pooled together. To precipitate the proteins, 7 mL of ice-cold acetone(aq) was added to each sample, followed by incubation at −20 °C overnight. After centrifugation at 10,000× *g* for 10 min, the supernatant was removed and the pellet was washed two times with 1 mL of 80% acetone each.

To the resulting pellets, 2 mL of digestion-buffer (50 mM TEAB, 1 mM MgCl2 in MS-grade water) and 2 mL of MS-grade water were added and the samples were resuspended by sonication (5 cycles at 50% power output, 5 min) on ice. Afterwards, the protein concentration of the samples was determined by conducting a Bradford assay.

A volume equivalent of 445 µg protein content was taken. For digestion, samples were processed in the same way as for the full-proteome analysis with the following amounts: TCEP(aq) (1M) was added to a final concentration of 41.8 mM, 255 µL of TEAB(aq) (1M) were added to neutralize the solution, iodoacetamide(aq) (1M) was added to a final concentration of 41.1 mM and 210 µL of TEAB(aq) (1M) were added to adjust the pH-value for digestion. Finally, 5 µg trypsin was added per sample (3 µg in case of the IA-triplicate samples).

After digestion, peptides were desalted by stage-tip purification (SDB-RPS material, see [43]) and concentrated to dryness on a speed-vac.

For each replicate, 50 µL MagReSyn^®^ magnetic Ti-IMAC beads (ReSyn Biosciences, Gauteng, South Africa) were prepared according to the manufacturer’s instructions. For loading, the dried peptides were dissolved in 100 µL loading-buffer (1M glycolic acid, 80% MeCN, 5% TFA in MS-grade water) and incubated for 20 min at room temperature with end-over-top mixing with the prepared Ti-IMAC beads. Unbound sample was removed by washing two times with 100 µL loading buffer for 30 s. Subsequently, the beads were incubated four times with 100 µL wash buffer (80% MeCN, 1% TFA in MS-grade water) for 2 min each. Phosphopeptides were eluted by incubation three times with 80 µL elution buffer (1% NH4OH in MS-grade water) for 15 min each.

After elution, phosphopeptide-containing samples were acidified with FA(aq), desalted by stage-tip purification (SDB-RPS material, see [43]) and concentrated to dryness on a speed-vac.

The phosphopeptide-samples were solved in 12 µL MS-solvent.

### 4.9. MS-Analysis of Phospho-Enriched Samples. 

The samples were analyzed with an UltiMate 3000 RSLCnano liquid chromatography system (Dionex, Thermo Fisher Scientific) attached to a Q Exactive HF mass spectrometer (Thermo Fisher Scientific). They were concentrated on a µ-precolumn cartridge (PepMap100, C18, 5 µM, 100 Å, size 300 µm i.d. × 5 mm (Dionex, Thermo Fisher Scientific)) and further processed on an in house packed analytical column (ReproSil-Pur 120 C18-AQ, C18, 1.9 µM, 120 Å (Dr. A Maisch GmbH), packed into a 75 µm i.d. × 150 mm fused silica picotip emitter with a 8 µm tip (New Objective, Littleton, MA, USA).

The samples were processed via a 120 min multi-step analytical separation at a flow rate of 300 nL/min and a column temperature of 30 °C. Only LC-MS grade solvents were used (solvent A: water + 0.1% formic acid; solvent B: acetonitrile + 0.1% formic acid). The gradient with percentages of solvent B was programmed in the following way: 1% for 3 min; from 1% to 6% in 2 min; from 6% to 34% in 85 min; from 34% to 60% in 10 min; from 60% to 85% in 5 min; 85% for 7 min; from 85% to 1% in 3 min; 1% for 5 min.

Per analysis, 11 µL of the full proteome samples and 11.5 µL of the phosphopeptide samples were injected.

Mass spectrometric analysis was done with a full mass scan in the mass range between m/z 300 and 1750 at a resolution of 120,000, an AGC target of 3e6 charges and a maximum ion injection time of 20 ms in profile mode. Following this survey scan, the 15 most intense ions were selected, fragmented and measured in profile mode with the following parameters: resolution of 15,000; AGC target of 2e5 charges; maximum ion injection time of 100 ms; isolation window of 2 m/z, with an offset of +0.3 m/z; stepped normalized HCD energy of 20%, 25% and 30%. Signals with an unrecognized charge state or a charge state of 1, 7, 8 or higher were not picked for fragmentation. To avoid supersampling of the peptides, signals were excluded from the analysis for 25 s after being selected for isolation and fragmentation. The peptide match setting was set to “-” and the exclude isotope setting was set to “on”.

Data analysis of the phosphor-enriched samples was performed with MaxQuant version 1.5.1.0 (MPI for Biochemistry, Martinsried) with the following settings: variable modifications of acetyl (protein N-term), oxidation (M) and phosphorylation (STY); fixed modification of carbamidomethyl on cysteines. FDR-levels were set to 0.01. Fast LFQ was performed for quantification. The “match between runs”-feature was enabled. A mus musculus FASTA (proteome-ID: UP000000589) was used from uniprot. Trypsin was set as the protease and a maximum of 2 missed cleavages and a minimal peptide length of seven was chosen. Further data analysis was performed with Perseus. To do so, LFQ intensities obtained from MaxQuant analysis were log2-transformed and only proteins identified in at least three of the retinal tissue samples were retained.

### 4.10. Statistical Analysis

One-way analysis of variance and unpaired Student’s t test were used to evaluate significant differences between multiple groups and two groups, respectively. Differences were considered statistically significant when *p* < 0.05. Data were analysed and graphed using the GraphPad Prism software (GraphPad Software).

## Supplementary Materials

The following are available online at https://www.mdpi.com/1422-0067/22/1/52/s1.

## Abbreviations

AAV	Adeno-associated virus
ACHM	Achromatopsia
cGMP	Cyclic guanosine monophosphate
DDR	DNA damage response
ER	Endoplasmic reticulum
MS	Mass spectrometry
UPR	Unfolded protein response
