# Supplementary material for: The cGMP-Dependent Protein Kinase 2 Contributes to Cone Photoreceptor Degeneration in the Cnga3-Deficient Mouse Model of Achromatopsia
Source: Int J Mol Sci. 2020 Dec 23;22(1):52. doi: 10.3390/ijms22010052 (PMC7793084; doi:10.3390/ijms22010052)
Supplement: Supplementary file 1 [file ijms-22-00052-s001.pdf]

Supplementary Figures

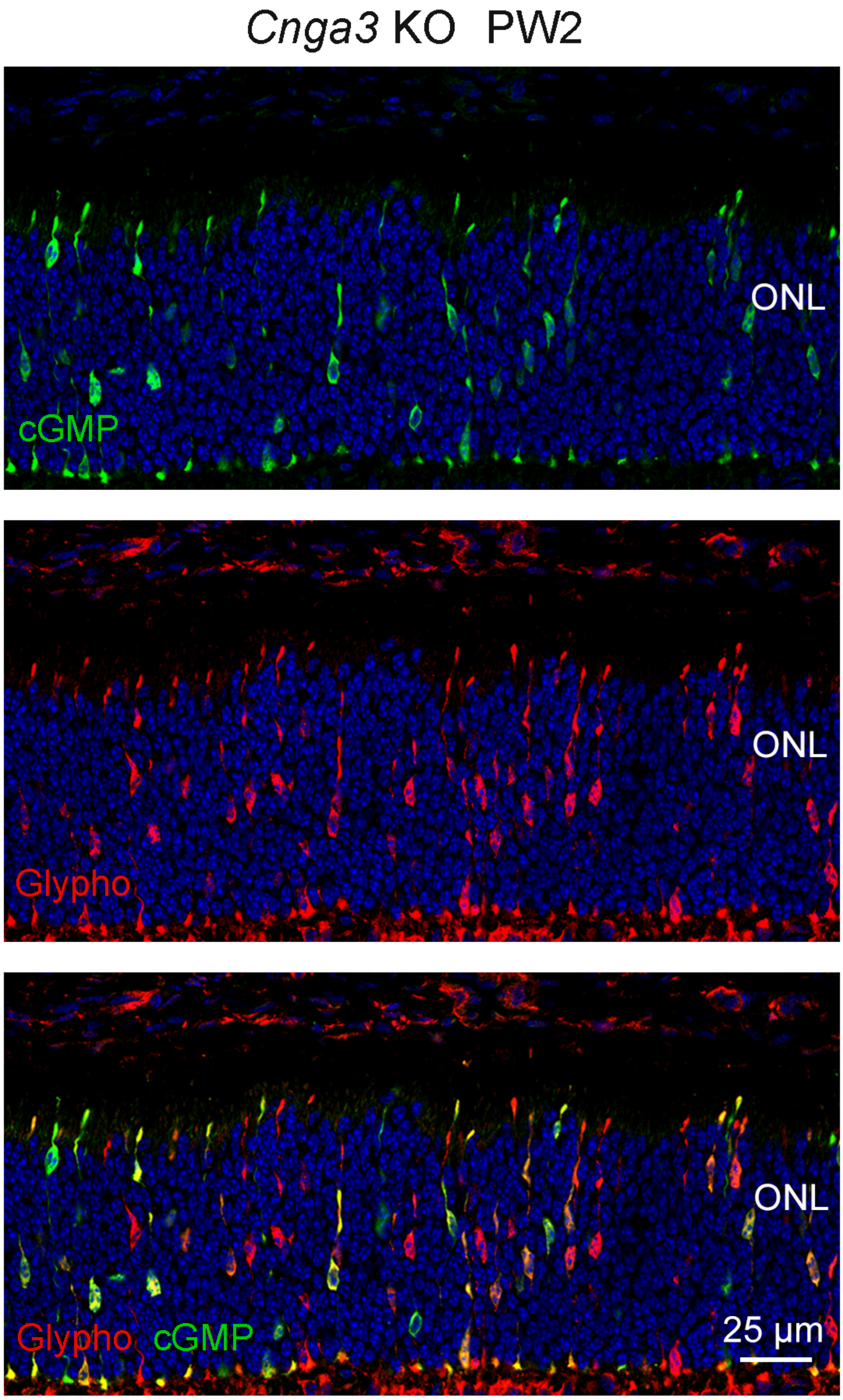

Supplementary Figure S1. Accumulation of cGMP in cone photoreceptors of *Cnga3* KO mice. Confocal scans from 2-week-old (PW2) *Cnga3* KO retinal cross-sections immunolabelled for cGMP (green) and glycogen phosphorylase (Glypho, red) to illustrate the exuberant levels of cGMP in affected cones. ONL, outer nuclear layer. Please note that due to delayed nuclear migration (see Ref. 6) cone cell bodies show aberrant localization.

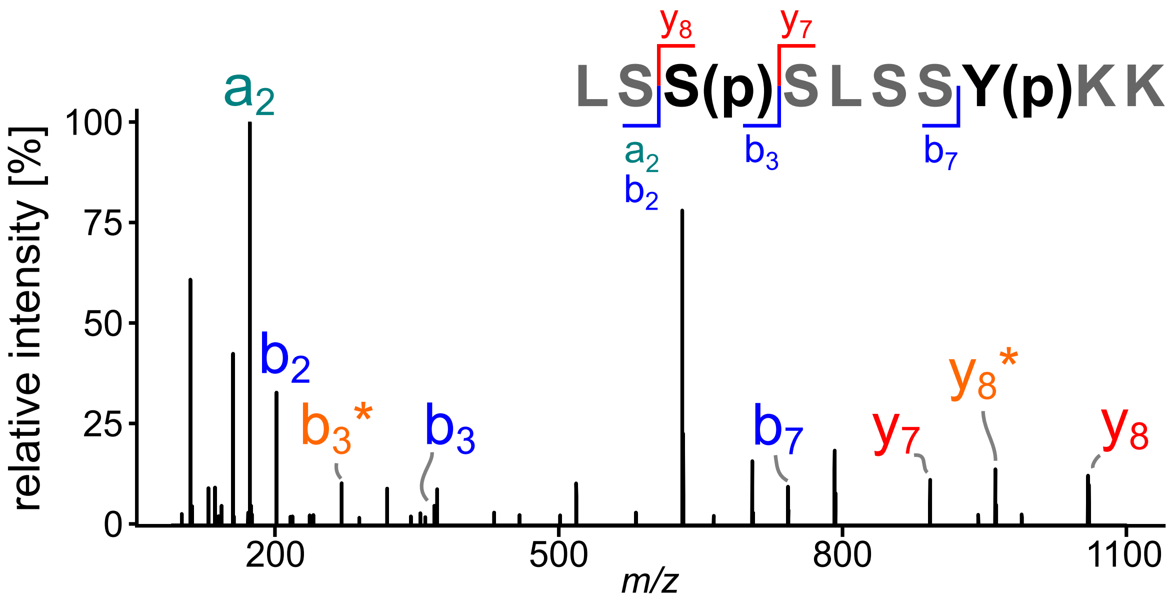

Supplementary Figure S2. Representative MS/MS spectrum of the Atr peptide LSS(p)SLSSY(p)KK, bearing two phosphorylations. Peaks utilized by MaxQuant for peptide identification are annotated. Phospho-localization is supported by the presence of two neutral loss peaks (orange, b3* and y8*)
